# A compressed large language model embedding dataset of ICD 10 CM descriptions

**DOI:** 10.1186/s12859-023-05597-2

**Published:** 2023-12-17

**Authors:** Michael J. Kane, Casey King, Denise Esserman, Nancy K. Latham, Erich J. Greene, David A. Ganz

**Affiliations:** 1 Department of Biostatistics, School of Public Health, Yale University, New Haven, USA; 2 The Jackson School of Global Affairs, Yale University, New Haven, USA; 3 US Healthcare and Life Sciences Microsoft, Redmond, USA; 4 Research Program in Men’s Health: Aging and Metabolism, Boston Claude D. Pepper Older Americans Independence Center for Function Promoting Therapies, Brigham and Women’s Hospital, Boston, USA; 5 Department of Medicine, VA Greater Los Angeles/UCLA, Los Angeles, USA

**Keywords:** Large language model, Autoencoder, ICD-10-CM, Electronic health records, EHR, NLP

## Abstract

This paper presents novel datasets providing numerical representations of ICD-10-CM codes by generating description embeddings using a large language model followed by a dimension reduction via autoencoder. The embeddings serve as informative input features for machine learning models by capturing relationships among categories and preserving inherent context information. The model generating the data was validated in two ways. First, the dimension reduction was validated using an autoencoder, and secondly, a supervised model was created to estimate the ICD-10-CM hierarchical categories. Results show that the dimension of the data can be reduced to as few as 10 dimensions while maintaining the ability to reproduce the original embeddings, with the fidelity decreasing as the reduced-dimension representation decreases. Multiple compression levels are provided, allowing users to choose as per their requirements, download and use without any other setup. The readily available datasets of ICD-10-CM codes are anticipated to be highly valuable for researchers in biomedical informatics, enabling more advanced analyses in the field. This approach has the potential to significantly improve the utility of ICD-10-CM codes in the biomedical domain.

**Supplementary Information:**

The online version contains supplementary material available at 10.1186/s12859-023-05597-2.

## Background

The International Classification of Diseases, 10th Revision, Clinical Modification (ICD-10-CM) [1] is a standardized classification system for categorizing diseases, disorders, and health conditions. ICD-10 was developed by the World Health Organization (WHO) and adapted for use in the United States as ICD-10-CM by the National Center for Health Statistics (NCHS) [2]. The standard plays a crucial role in the analysis of electronic medical records (EMRs) or electronic health records (EHRs) for several reasons:

1. Consistency and Standardization: The ICD-10-CM allows for a consistent and standardized method of coding and documenting medical conditions across healthcare providers and facilities. This helps to ensure accurate and uniform data exchange, analysis, and comparison.
2. Data Analysis and Research: The ICD-10-CM codes can be used to analyze patient data for clinical research, epidemiological studies, and public health surveillance. It helps to identify trends and patterns in diseases, monitor the effectiveness of treatments, and develop better prevention and management strategies.
3. Quality Measurement and Improvement: ICD-10-CM codes can be used to evaluate the quality of care provided by healthcare facilities, monitor patient outcomes and identify areas for improvement. This information can be used to enhance the overall healthcare delivery system.
4. Reimbursement and Billing: ICD-10-CM codes play a vital role in healthcare reimbursement by providing a standardized method to classify and report medical conditions. Insurance companies and other payers use these codes to determine appropriate payments for medical services rendered.
5. Health Policy and Planning: ICD-10-CM codes help health authorities and policymakers to identify population health needs, allocate resources, and develop targeted healthcare policies and interventions.

While ICD-10-CM codes do provide a consistent and comprehensive set of categories, their incorporation into statistical and machine learning analyses can be challenging for several reasons. First, in the 2019 version of the standard, there were 71,932 categories, increasing to 72,184 categories in 2020; 72,616 categories in 2021; and 72,750 categories in 2022. As a result, analyses using these codes, where the set of codes is not restricted to a smaller set, must take into account their high dimensionality or will require a large number of training samples in order to fit consistent models. Second, categorical variables are usually incorporated into analyses with a contrast encoding such as treatment, helmert, etc. Contrast numeric representations are orthogonal or, under appropriate statistical assumptions, independent with respect to their categories. However, ICD-10-CM codes represent a hierarchical structure, where codes are organized into chapters, blocks, and categories based on the type and anatomical location of the diseases or conditions. Applying traditional contrast encoding methods may not fully capture this hierarchical information, potentially resulting in a loss of valuable context and relationships between codes.

Researchers have considered alternative encoding methods or feature extraction techniques that can better represent the hierarchical structure of ICD-10-CM codes. However, incorporating both hierarchical structure and other contextual information in a general way can be difficult. The previous generation of word embeddings, which provide vector-encodings of words, were shown effective for these types of tasks, with models like med2vec [3] providing improved abilities to predict patient mortality; inpatient2vec [4] to predict clinical events; EHR2Vec [5] to help analyze sequences of patient visits; and cui2vec [6] to learn medical concepts based on multimodal clinical data. These models have been foundational in advancing the capabilities of machine learning models in understanding and generating human language. These models are shallow, two-layer neural networks that are trained to reconstruct linguistic contexts of words. Word embeddings produced by Word2Vec [7] and previously mentioned variants, provide vector representations of words in a continuous vector space where semantically similar words are mapped to nearby points. Within this class of models there are two main training algorithms: Continuous Bag of Words and Skip-Gram models [8]. The former predicts target words (e.g., ’apple’) from source context words (’the fruit’). The latter performs the inverse and predicts source context words from the target words, and tends to perform better on larger datasets and produces higher-quality embeddings for less frequent words.

Despite their advantages, word embeddings also have certain limitations. First, word embeddings are typically generated at the word or code level, and while word embeddings can capture semantic similarities, they ofen struggle to represent hierarchical representations like those found in ICD-10-CM codes. Second, traditional word embeddings generate a single vector for each word regardless of context. This means that the same code can have different meanings depending on where and when it is used. This is something these models do not capture. Third, word embeddings can have difficulty handling rare codes. Word embeddings typically require a sufficient number of training samples to learn meaningful representations. For rarely used ICD-10-CM codes, the learned embedding might not be reliable. Fourth, traditional word embeddings provide static representations and do not change over time. However, in healthcare, the meaning and usages of certain codes can evolve, and these models cannot capture dynamic changes. Finally, the quality and representativeness of the word embeddings depend on the training data used to generate them. If the training data does not adequately cover the entire spectrum of medical conditions or encounters, the embeddings may not capture all relevant relationships or information.

The Transformer model [9] is a more recent architecture primarily designed for handling sequences, and it has become the foundation for many recent models in natural language processing, including the Bidirectional Encoder Representation Transformer (BERT) [10], the Generative Pre-Trained Transformer (GPT) [11], and the Text-to-Text-Transfer-Transformer T5 [12]. The Transformer model’s main innovation is its self-attention mechanism, which weighs input elements dynamically based on their content and relationship. This allows the model to focus on different parts of the input for different tasks or even different parts of the same task.

These models fall under the category of Large language models (LLMs) and address some of the shortcomings of traditional word embeddings through a combination of advanced techniques and architectures. Unlike traditional word embeddings that generate static representations, LLMs generate contextualized embeddings. These embeddings take into account the surrounding words or tokens, allowing for a more nuanced representation of words and codes in different contexts. This helps in capturing the semantic relationships between codes more effectively. These models are pre-trained on vast amounts of text data, allowing them to learn general language representations before being fine-tuned for specific tasks. This pre-training enables the models to leverage existing knowledge and adapt more effectively to new tasks, even with limited task-specific data. LLMs can be incrementally updated or fine-tuned with new data, allowing them to adapt to evolving medical knowledge and practices more effectively than static word embeddings. And, while not explicitly designed for hierarchical data like ICD-10-CM codes, LLMs can implicitly capture aspects of structured hierarchical relationships through their deep architectures and the context in which codes appear. This can help capture different levels of granularity and relationships between codes more effectively than traditional word embeddings.

Vector embeddings attempt to optimize the conditional probability of observing the actual output word given an input word (or vice versa, depending on the variant used). For instance, in the skip-gram variant, given a word $w_i$ and a context word $w_j$, the model is trained to maximize the following

$$\begin{aligned} P(w_j|w_i) = \frac{e^{v^T{w_j}^T v{w_i}}}{\sum _k e^{v^T{w_k}^T v{w_i}}} \end{aligned}$$

where $v_w$ and $v'_w$ represent the “input” and “output” vector representations of a word w, and the summation in the denominator is over all words in the vocabulary. The vectors $v_w$ and $v'_w$ are the word embeddings learned by a similarity model.

LLM models also start by converting each word into an initial word embedding using an embedding matrix. However, these initial embeddings are then updated based on the context of the word. This is done by passing the embeddings through several layers of a transformer model, which uses self-attention mechanisms. The output of the transformer is a contextual embedding for each word. Mathematically, the self-attention mechanism can be represented as

$$\begin{aligned} \text {Attention}(Q, K, V) = \text {softmax}(QK^T/\sqrt{d}) V \end{aligned}$$

where *Q*, *K*, and *V* represent the query, key, and value matrices, which are derived from the input embeddings. The softmax function ensures that the weights of different words sum to 1, and the $\sqrt{d}$ in the denominator is a scaling factor that improves the stability of the gradients during training. The resulting matrix product is a weighted sum of the value vectors, where the weights depend on the similarity between the query and key vectors.

To generate an embedding for a sentence or description, one common approach is to take the average of the contextual embeddings of the words in the sentence:

$$\begin{aligned} E(D) = \frac{1}{n } \sum E(w_i) \end{aligned}$$

Here, *E*(*D*) is the embedding for the description, $E(w_i)$ is the contextual embedding for word $w_i$, and the sum is over all words in the description.

The key difference between the two methods is that vector embeddings generate a single, static embedding for each word, while LLMs generate a dynamic, context-dependent embedding. This allows an LLM to capture nuances in meaning that cannot be represented with static embeddings.

There are several BERT or similar transformer-based biomedical models that can been used to generate embeddings for medical corpuses including ClinicalBERT [13, 14], BioBERT [15], and Med-BERT [16], but to our knowledge none of the current literature includes the applications of these models specifically for the purpose of generating embeddings for ICD-10-CM code that can be consumed as readily available data sets. These data sets represent a valuable resource for practioners who are interested in an information-rich representation of those codes, without needing to acquire models, embed data, and process them.

This paper describes data sets provided as .csv files, which are available online in the form of a crosswalk from ICD-10-CM codes to embeddings (a numeric vector of values), based on their descriptions. A sample of five descriptions and their embeddings are provided in Additional file 1. The embeddings were generated using the BioGPT LLM [17],which was trained on the biomedical literature including PubMed [18], PubMed Central [19], and clinical notes from MIMIC-III [20]. This model was shown to be superior at encoding context and relational information than competitors in the medical domain. Since the dimension of the embedding LLM is relatively high (42384), we provide dimension-reduced versions in 1000, 100, 50, and 10 dimensions. The model generating the data was validated in two ways. The first way validates the dimension reduction. The embedding data were compressed using an auto-encoder. The out-of-sample accuracy of a validation set is examined as well as the performance of the model for other versions (by year) of the ICD-10-CM specification. Our results show that we can reduce the dimension of the data down to as few as 10 dimensions while maintaining the ability to reproduce the original embeddings, with the fidelity decreasing as the reduced-dimension representation decreases. The second way validates the conceptual representation by creating a supervised model to estimate the ICD-10-CM hierarchical categories. Again, we see as the dimension of the compressed representation decreases, the model accuracy decreases. Since multiple compression levels are provided, users are free to choose whichever suits their needs, allowing them to trade off accuracy for dimensionality.

The paper proceeds as follows. The next section provides a high-level description of the BioGPT and the embedding along with the construction of the autoencoder used to reduce the dimension of the embedding representation. That section then provides validation for both the dimension reduction as well as the representation. The third section provides an example of how to use the dataset to cluster ICD-10-CM codes using the R programming environment [21]. The final section provides a broader look at the incorporation of LLM approaches to these types of data.

The data sets and code to generate them are available in a public repository on Github.^1^ The data are licensed under the Creative Commons Attribution NonCommercial ShareAlike 4.0 International License.^2^ The code is licensed under GPL-v2.^3^

## Construction and content

The provided data are generated by embedding ICD-10-CM descriptions using the BioGPT-Large model, which comprises 1.5 billion parameters and is accessible via the Hugging Face model repository,^4^ and then performing a dimension reduction using an autoencoder. The embedding process involves tokenizing textual phrases into tokens (words, subwords, or characters) and mapping them to unique vocabulary IDs. Token IDs are passed through an embedding layer, resulting in a sequence of continuous embedding vectors. Positional encodings are added elementwise to these vectors, enabling the model to capture token order and relative positions. The embeddings are then contextualized by passing them through the model’s layers. An attention mask selectively controls information flow in the attention mechanism, allowing the model to weigh the importance of input tokens when generating contextualized embeddings in a 42384-dimension space.

The embedding is then compressed using an autoencoder. The autoencoder used here is a series of fully connected layers where the number of hidden nodes is approximately one order of magnitude smaller than the previous layer and then an order of magnitude larger until the output layer. For example, the autoencoder compressing to 10 dimensions has layers of size 42384, 1000, 100, 50, 10, 50, 100, 1000, 42384. Models whose dimension is large use the same structure while retaining only the appropriate layers. A practioner who would like to make use of these embeddings for their own modeling task, can download these data, substituting the embedding values for the ICD 10 representation. The values are information-rich and will be useful in a variety of supervised and unsupervised tasks involving medical research.

### Validating the dimension reduction

The autoencoder compressing the LLM embedding was fit on the 2019 ICD-10-CM descriptions for 20 epochs, with batch sizes 64, 128, and 256. The mean-square error loss between the embedding and autoencoder estimate, and a validation data set comprised of random subset of 10% of the samples. The model performance is shown in Table 1. Based on these results the models with the best validation loss for each of the compressed embedding dimensions selected for further validation and eventual distribution. In addition, benchmarking the validation loss serves two purposes. First, it establishes a relative measure of performance quantifying the compression loss and allowing us to pick the best set of model parameters to generate the embedding data. Second, the validation loss in particular quantifies how much loss is incurred by new ICD-10-CM codes showing that the loss is comparable to, and often less than, the error in the training data.

In addition to the 2019 validation, the models selected for distribution were tested on the 2020-2022 data sets to ensure their performance is comparable over years. The results are shown in Table 2. It should be noted that the ICD-10-CM codes do not vary much from one year to the next, so we should not expect large differences. As expected, the mean square error and coefficients of determination are similar to the 2019 data. For a given embedding dimension it can be seen that neither the coefficient of determination nor the mean square error change significantly over years indicating that the same autoencoder could likely be used in subsequent years, while incurring similar loss. This also implies that an incremental approach could be taken in subsequent years when regenerating the embeddings where only new codes would need to be processed.

**Table 1:** The autoencoder parameters and performance ordered by increasing validation loss

Embedding dimension	Batch size	Training loss	Validation loss
100	64	0.534	0.339
100	128	0.487	0.381
50	256	0.403	0.392
1000	64	0.542	0.402
100	256	0.556	0.444
1000	128	1.073	0.486
10	256	0.599	0.594
10	128	0.628	0.609
10	64	0.679	0.641
50	64	1.134	0.699
1000	256	30.435	0.803
50	128	1.053	0.894

**Table 2:** The autoencoder validation performance ordered by year

Year of Published ICD-10-CM Code	Embedding dimension	Mean square error	Coef. of determination
2019	10	0.593	0.086
2019	50	0.388	0.056
2019	100	0.336	0.049
2019	1000	0.400	0.058
2020	10	0.593	0.086
2020	50	0.388	0.056
2020	100	0.336	0.049
2020	1000	0.400	0.058
2021	10	0.594	0.086
2021	50	0.389	0.056
2021	100	0.337	0.049
2021	1000	0.401	0.058
2022	10	0.595	0.086
2022	50	0.390	0.056
2022	100	0.338	0.049
2022	1000	0.402	0.058

### Validating the embedding representation

As a final step in the validation process, we use the fact that in addition to the description, the ICD-10-CM codes themselves carry hierarchical information, which can be used to ensure that conceptual relationships are preserved in the compressed embeddings. In particular, the leading letter and two numeric values categorize codes For example, codes A00-B99 correspond to infectious and parasitic diseases, C00-D49 correspond to neoplasms, etc. There are a total of 22 codes. The full table of categories is provided in the Additional file 1. We can therefore ensure that at least some of the relevant relationships are preserved in the compressed embedding representation by confirming that the categories can be estimated at a rate higher than chance using a supervised model. Furthermore, we can quantify how much relevant predictive information is lost in lower-dimensional representations.

The training data consists of a one-hot encoding of the ICD-10-CM categories as the dependent variable and the compressed embedding values as the values. The model consists of two hidden layers with 100 nodes each. The loss function selected was categorical cross-entropy. The model was trained using 30 epochs and a validation data set comprised of 10% of samples, chosen at random.

To contextualize the results, we fit the same model to four BERT embeddings that have also been trained on biomedical corpuses. The first, MedBERT [22] was trained with 57.46M tokens collected from biomedical-related data sources and biomedical-related articles from Wikipedia. The second, PubMedBERT-MS-MARCO [23] was first trained on Pubmed abstracts and full texts and then fine-tuned using the MS-MARCO data set [24] to be optimized for information retrieval task in the medical/health text domain. The third, SapBERT-PubMedBERT, was first trained on Pubmed abstracts and text, and then fine-tuned semantic relationships between relevant medical entities using UMLS [25] biomedical ontologies. The fourth, ClinBERT [13] was initialized from BERT. Then the training followed the principle of masked language model, in which given a piece of text, we randomly replace some tokens by MASKs, special tokens for masking, and then require the model to predict the original tokens via contextual text.

The performance in terms of both the out-of-sample accuracy and the out-of-sample balanced accuracy [26] is shown in Table 3. The goal in presenting these results is not to necessarily to maximize the prediction accuracy. Rather, it is to show that the embedding retains the hierarchical information in the ICD-10-CM codes. Some of the codes correspond to conditions that could be classified in several ways, and as a result coding for at least some of the conditions might be considered non-systematic. Based on this criterion, we can conclude the embedding does retain much of the structural and conceptual information denoted in the descriptions, at least in terms of mapping to key categories of diseases and conditions.

The table provides two main results. First, the models using the BioGPT compressed representation significantly outperform models based on BERT models with the the former outperforming the latter, even after compressing the BioGPT embedding to 10 dimensions. Second, for the BioGPT compressed embeddings, great compression of the data correpsonds to a decrease in the predictive information in the data, as measured by the accuracy.

Since the ICD-CM-10 codes are themselves heirarchical with the category codes being the broadest category it is worth pointing out that these results imply that some aspect of the code hierarchy is preserved in the embedding. However, the extent to which this hierarchy can be fully recovered remains an area of limited understanding. A potential avenue for future work could entail exploring the feasibility of mapping the embedding space to established ontologies, such as the UMLS.

**Table 3:** The supervised models’ performance ordered by decreasing balanced accuracy

Model	Embedding dimension	Accuracy	Balanced accuracy
BioGPT Compressed	1000	0.960	0.927
BioGPT Compressed	100	0.935	0.891
BioGPT Compressed	50	0.925	0.873
BioGPT Compressed	10	0.815	0.698
ClinicalBERT	768	0.200	0.634
PubMedBERT-MS-MARCO	768	0.158	0.629
SapBERT-PubMedBERT	768	0.159	0.616
MedBERT	768	0.171	0.613

## Conclusions

This paper presents novel datasets offering numerical representations of ICD-10-CM codes by generating description embeddings using a large language model and applying autoencoders for dimensionality reduction. The approach is versatile, capable of handling categorical variables with numerous categories across various domains. By capturing relationships among categories and preserving inherent information, the embeddings serve as informative input features for machine learning models. The readily available datasets are anticipated to be highly valuable for researchers incorporating ICD-10-CM codes into their analyses, retaining contextual information. This approach has the potential to significantly improve the utility of ICD-10-CM codes in biomedical informatics and enable more advanced analyses in the field. Data analysts can easily incorporate them into their own analyses by substituting the embedding values for other, lower-information representations including the categorical ones described above to derive the benefits of the conceptual information encoded in their embedding. Future work will address some of the challenges of capturing hierarchical structure in ICD-10-CM coding systems, experimenting with Ontology-based methods, hierarchical clustering, hierarchial autoencoding, graph neural networks and incorporating hierarchical information in training.

While this approach is effective, there are some challenges of which we should be aware. While not insurmountable, they are as follows:

1. Interpretability: A significant challenge in machine learning, particularly with complex models like large language models and autoencoders, is interpretability. In healthcare, the ability to understand and explain why a model makes a particular prediction is crucial. This could impact patient trust, clinician adoption, and even legal and regulatory compliance. Techniques like LIME (Local Interpretable Model-Agnostic Explanations) or SHAP (SHapley Additive exPlanations) can be used to improve interpretability, but they do not provide perfect solutions and can be computationally expensive.
2. Overfitting: Overfitting is a common issue in machine learning where a model learns the training data too well and performs poorly on unseen data. This can be particularly problematic in healthcare, where the stakes are high. Techniques such as cross-validation, regularization, or dropout layers can be used to prevent overfitting.
3. Data Privacy: Patient data is highly sensitive, and its usage is strictly regulated (e.g., by laws like HIPAA in the US). Even if the data used to generate the embeddings is anonymized, the model must be carefully designed and used to avoid potential privacy leaks.
4. Generalizability: A model trained on one dataset may not perform well on another due to differences in population characteristics, data collection methods, etc. Ensuring that models generalize well across different settings is a significant challenge.
5. Quality of Input Data: The quality of the embeddings depends heavily on the quality of the input data. If the descriptions associated with the ICD-10-CM codes are inaccurate or not comprehensive, the resulting embeddings may also be flawed. This is a fundamental issue in any data-driven approach: “garbage in, garbage out.”
6. Capturing Hierarchical Structure: The ICD-10-CM coding system has a hierarchical structure where certain codes are nested within broader categories. While embeddings generated from code descriptions may capture semantic meaning, they might not adhere to an explicit hierarchical imposed by an ontology like UMLS.

## Example use of the ICD-10-CM embedding data

To illustrate the utility of the data, we present a simple example of how one might use the embedding information in the R programming environment and making use of the dplyr [27], ggplot2 [28], readr [29], Rtsne [30], and stringr [31] packages. Suppose we would like to visualize the ICD-10-CM codes beginning with G (diseases of the nervous system), I (diseases of the circulatory system), J (diseases of the respiratory system), and K (diseases of the digestive system) to better understand the contextual relationships between these categories or specific conditions in the the 50-dimensional embedding. For convenience, the projects page includes an .rds file containing the available embeddings along with their URLs, which can be retrieved from the R console. The code categories can then be visualized by performing another dimension reduction (in this case we will use the Rtsne package), to 2 dimensions that can be presented as a scatter plot.
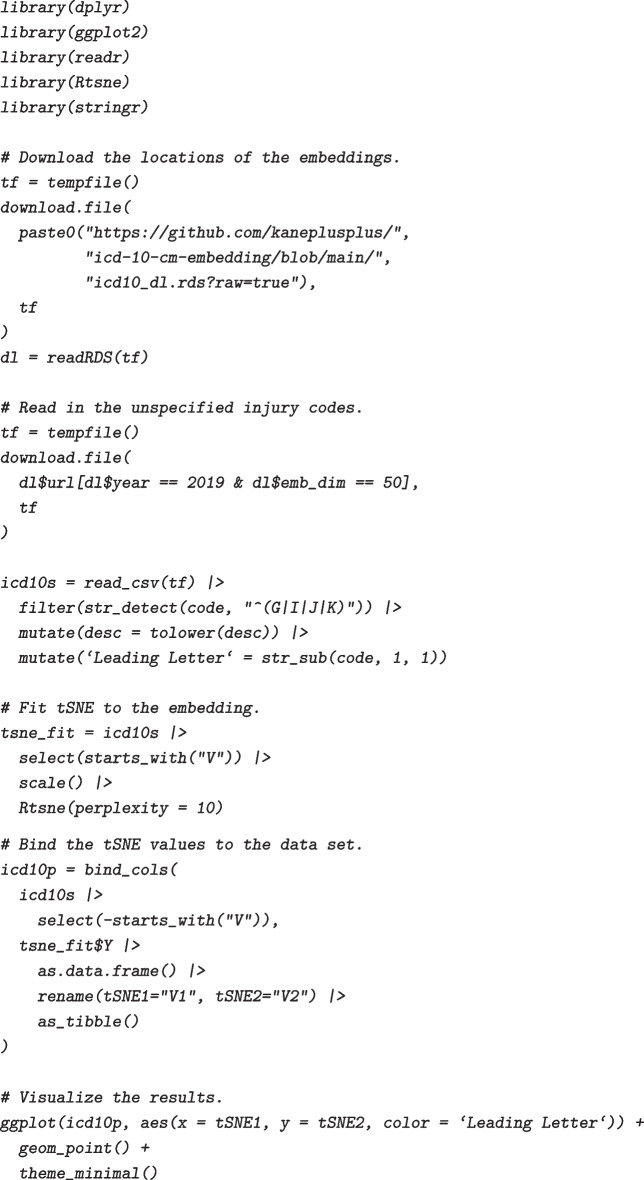


**Fig. 1:**
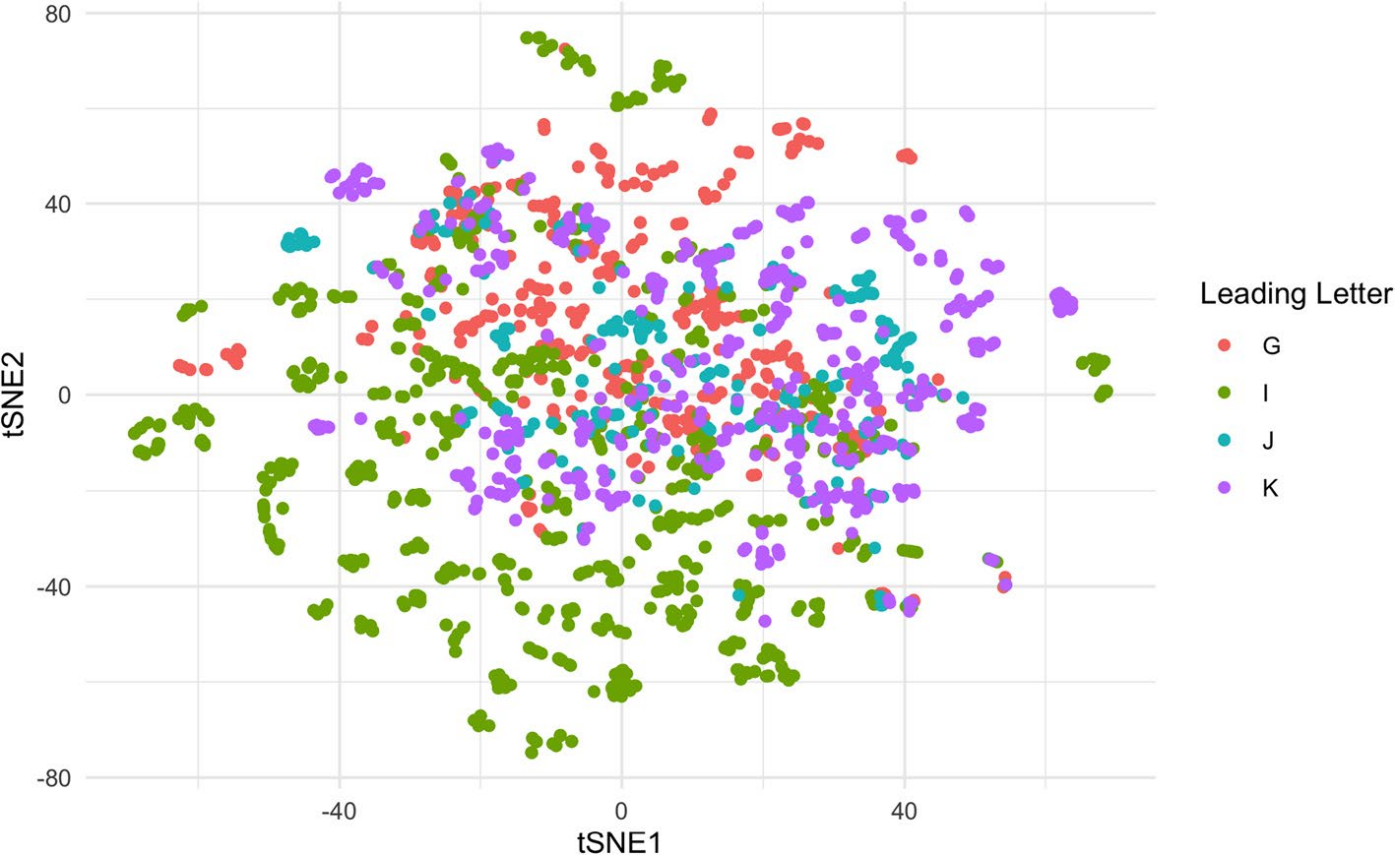
The tSNE plot of the codes

The output visualization is presented in Fig. 1 and shows that a subset of the circulatory diseases (I) and nervous system diseases (G) are well-differentiated from other conditions. It also shows overlap between other conditions related to K (digestive diseases), J (respiratory diseases), and I (circulatory).

### Supplementary Information

**Additional file 1.** Example embedded documents visualized using T-SNE.

## Data Availability

All data presented here along with documentation for reproducing presented materials is available at https://github.com/kaneplusplus/icd-10-cm-embedding.

## Footnotes

1 https://github.com/kaneplusplus/icd-10-cm-embedding.

2 https://creativecommons.org/licenses/by-nc-sa/4.0/legalcode.

3 https://www.gnu.org/licenses/old-licenses/gpl-2.0.en.html.

4 https://hugginface.co.

## Code availability

All code presented here along with documentation for reproducing presented materials is available at https://github.com/kaneplusplus/icd-10-cm-embedding.
