# Supplementary material for: A compressed large language model embedding dataset of ICD 10 CM descriptions
Source: BMC Bioinformatics. 2023 Dec 17;24:482. doi: 10.1186/s12859-023-05597-2 (PMC10726612; doi:10.1186/s12859-023-05597-2)
Supplement: Supplementary file 1 — Additional file 1. Example embedded documents visualized using T-SNE. [file 12859_2023_5597_MOESM1_ESM.pdf]

**Supplementary Materials**

1.2 ICD-10-CM Category Codes

| Code | Description |
|---|---|
| S3559XS | Injury of other iliac blood vessels, sequela |
| M12262 | Villonodular synovitis (pigmented), left knee |
| S40011S | Contusion of right shoulder, sequela |
| K284 | Chronic or unspecified gastrojejunal ulcer with hemorrhage |
| M90632 | Osteitis deformans in neoplastic diseases, left forearm |

ICD-10-CM Category Codes

| First Code | Last Code | Code Description |
|---|---|---|
| A00 | B99 | Certain infectious and parasitic diseases |
| C00 | D49 | Neoplasms |
| D50 | D89 | Diseases of the blood and blood-forming organs and certain disorders involving the immune mechanism |
| E00 | E89 | Endocrine, nutritional and metabolic diseases |
| F01 | F99 | Mental, Behavioral and Neurodevelopmental disorders |
| G00 | G99 | Diseases of the nervous system |
| H00 | H59 | Diseases of the eye and adnexa |
| H60 | H95 | Diseases of the ear and mastoid process |
| I00 | I99 | Diseases of the circulatory system |
| J00 | J99 | Diseases of the respiratory system |
| K00 | K95 | Diseases of the digestive system |
| L00 | L99 | Diseases of the skin and subcutaneous tissue |
| M00 | M99 | Diseases of the musculoskeletal system and connective tissue |
| N00 | N99 | Diseases of the genitourinary system |
| O00 | O9A | Pregnancy, childbirth and the puerperium |
| P00 | P96 | Certain conditions originating in the perinatal period |
| Q00 | Q99 | Congenital malformations, deformations and chromosomal abnormalities |
| R00 | R99 | Symptoms, signs and abnormal clinical and laboratory findings, not elsewhere classified |
| S00 | T88 | Injury, poisoning and certain other consequences of external causes |
| U00 | U85 | Codes for special purposes |
| V00 | Y99 | External causes of morbidity |
| Z00 | Z99 | Factors influencing health status and contact with health services |
